# Measuring self-esteem after spinal cord injury: Development, validation and psychometric characteristics of the SCI-QOL Self-esteem item bank and short form

**DOI:** 10.1179/2045772315Y.0000000014

**Published:** 2015-05

**Authors:** Claire Z. Kalpakjian, Denise G. Tate, Pamela A. Kisala, David S. Tulsky

**Affiliations:** 1 Department of Physical Medicine and Rehabilitation, University of Michigan Medical School, University of Michigan, Ann Arbor, MI, USA; 2 Department of Physical Therapy, College of Health Sciences, University of Delaware, Newark, DE, USA; 3 Kessler Foundation Research Center, West Orange, NJ, USA

**Keywords:** Self-concept, Self-assessment, Spinal cord injuries, Patient outcomes assessment, Quality of life, Psychometrics, Self-esteem

## Abstract

**Objective:**

To describe the development and psychometric properties of the Spinal Cord Injury-Quality of Life (SCI-QOL) Self-esteem item bank.

**Design:**

Using a mixed-methods design, we developed and tested a self-esteem item bank through the use of focus groups with individuals with SCI and clinicians with expertise in SCI, cognitive interviews, and item-response theory- (IRT) based analytic approaches, including tests of model fit, differential item functioning (DIF) and precision.

**Setting:**

We tested a pool of 30 items at several medical institutions across the United States, including the University of Michigan, Kessler Foundation, the Rehabilitation Institute of Chicago, the University of Washington, Craig Hospital, and the James J. Peters/Bronx Department of Veterans Affairs hospital.

**Participants:**

A total of 717 individuals with SCI completed the self-esteem items.

**Results:**

A unidimensional model was observed (CFI = 0.946; RMSEA = 0.087) and measurement precision was good (theta range between −2.7 and 0.7). Eleven items were flagged for DIF; however, effect sizes were negligible with little practical impact on score estimates. The final calibrated item bank resulted in 23 retained items.

**Conclusion:**

This study indicates that the SCI-QOL Self-esteem item bank represents a psychometrically robust measurement tool. Short form items are also suggested and computer adaptive tests are available.

## Introduction

The concept of *self-esteem* is characterized by emotional, evaluative, and cognitive perceptions of the self. Self-esteem is one of the most popular psychological concepts and has been extensively studied, embedding itself in social and popular consciousness.^1^ In 1890, William James first introduced the concept of self-esteem in his *Principles of Psychology*^2^ as the result of a splitting of ourselves into a ‘knower self’ and ‘known self.’ Rosenberg later commented^3^ on the unique ability to be the object of one's own evaluation, reflecting the duality that James considered the basis of self-esteem. Most simply, self-esteem can be thought of as a favorable or unfavorable attitude towards oneself.

Low self-esteem has been robustly associated with depression across populations^4^ and many other positive and negative psychological states.^5^ Threats to self-esteem can be internal and external. Public failure is a common external threat; internal, self-generated threats to self-esteem are characterized by cognitions and introspection.^6^ Self-esteem also has been to shown to act as a buffer for emotional reactions to negative life events, mitigating their impact on emotional distress.^7^

Although not as widely studied in the context of disability and rehabilitation, there is evidence that illness and disability can negatively impact self-esteem^8–10^ and that, conversely, low self-esteem can exacerbate symptoms, stress, and negative mood.^11^ There is also evidence that inpatient rehabilitation may improve self-esteem.^12,13^ There is some evidence that self-esteem also plays a role in the well-being of persons with spinal cord injury (SCI). A 2012 systematic review of psychological resources in persons with SCI showed positive associations between self-esteem and well-being, mental health, community participation, mastery, hope and more effective coping.^14^ This review also suggested that self-esteem is often compromised by SCI, but with some evidence for restoration.^14^ Earlier work also showed a perceived loss of self-esteem after injury and may rebound over time with a low period in the second year post-SCI.^15^ A loss of self-esteem soon after a disabling event, and subsequent rebound, have also been observed in persons with acute stroke.^13^ Another recent systematic review suggested that related concepts of self-efficacy and self-worth are also strongly and consistently related to quality of life after SCI.^16^ In persons with SCI, high levels of self-esteem and self-efficacy also have been associated with community participation, above and beyond the influence of factors such as anxiety and depression, pain, social support, or coping style.^17^

Social comparison also plays a role in self-esteem, particularly among those with high levels of stress and uncertainty.^18^ Social comparison theory centers on the belief that individuals compare themselves to others to reduce uncertainty about their opinions and abilities and as a means to define themselves.^19^ Early work in SCI and adjustment suggested that persons with SCI compared themselves most often to people in general, far less to others with SCI, and even less so to healthy individuals.^20^ In contrast, a more recent study of social comparison in SCI suggested that individuals compared themselves to healthy individuals as often as others with SCI; those who did so were more likely to also have high levels of stress and uncertainty.^21^

Self-esteem and social comparison, in particular, have also been linked to coping strategies in various populations with chronic illness.^22^ In particular, *upward contrast* (negative feelings towards those who are better off) and *downward identification* (fear of becoming like others who are worse off) have been significantly associated with greater depression in persons with SCI.^21^ Coping strategies were also significantly associated with dimensions of self-esteem, such as wishful thinking associated with upward identification (hope to be like others who are better off) or blaming others with downward identification.

Measuring self-esteem is generally accomplished in one of two ways.^23^ The first is indirectly by: (1) measuring traits theorized to comprise self-esteem and then aggregating item-level or trait-level data; (2) using factor analytic techniques; or (3) modeling the higher-order self-esteem construct with item- and trait-level data. The second is to measure the construct directly. One of the most commonly used measures is the Rosenberg Self-esteem Scale^24^ and a number of other ad hoc scales have been created from various data and items from other scales, though these are inconsistently validated. Typically, measures of self-esteem traits or direct measures of the construct are general and not situation-specific. Such measures reflect the construct of self-esteem as a global evaluation of oneself.^23^

Despite the popularity of self-esteem as a psychological concept and its relevance for understanding adaption and adjustment processes after SCI, there are currently no SCI-specific measures to assess the construct of self-esteem. Established measures of self-esteem are designed for those who have not necessarily experienced a life-altering event. To address important questions about adaptation and coping after SCI, self-esteem measures require greater specificity. Thus, the purpose of this paper is to present findings from the development and psychometric calibration of the SCI-QOL Self-esteem item banks and short forms. An overview of the entire SCI-QOL measurement system is provided in the introductory article to this issue.^25^

## Methods

This study was approved by all participating sites' Institutional Review Boards. The first study activity was to develop and refine a self-esteem item pool. Next, self-esteem items were administered to a large sample of people with SCI using a computerized data collection platform and interview format, so that each question was read to the respondent by a trained interviewer and responses were directly entered into the database. Each of these steps is described in detail in Tulsky *et al*. (this issue)^26^ and is also outlined briefly in the section below.

### Development of a self-esteem item pool

To develop the self-esteem item bank, we began by identifying candidate items from our initial pilot work, which included individual, semi-structured interviews and focus groups with individuals with SCI and SCI clinicians (see Tulsky *et al*.^27^ for a full description). From these data, we developed a set of 17 preliminary items related to self-esteem. Specific phrases or concepts were drawn from the interviews and focus group transcripts and converted into 17 additional ‘new’ items. For example, a focus group participant with tetraplegia stated, ‘*I got hurt up here…You know they call this the quad pod. Right here. Just – and I know I'm not heavy for a [person with] spinal cord [injury] – I only went up one size and I haven't gained a pound since my injury…*’ and from that quote we drafted the item, ‘I was unhappy with changes in my posture.’ Five more items were drawn from the Quality of Life in Neurological Disorders (Neuro-QOL) measurement system with retention of the exact wording of the item, with the exception of changing, with permission, the word ‘illness’ to ‘injury’ Some items were redundant with the new items created from interviews and focus groups. In these cases, if the overlap was deemed sufficient, the new items were dropped in favor of the Neuro-QOL items to maintain consistency.

The initial 39 items then underwent Expert Item Review (EIR),^28^ a method whereby several project co-investigators reviewed each item for relevance and clarity and made suggestions for revisions and deletions. Based on EIR feedback, 25 items were retained in the preliminary self-esteem item pool. Preliminary items then underwent an additional phase of item review and modification by members of the investigative team. Items were arranged on a hierarchy of ‘difficulty’, from items indicating the lowest degree of self-esteem to the highest degree of self-esteem. Team members removed redundant items where there was oversaturation in the middle range of the hierarchy, and suggested new items to fill gaps in content coverage. During this phase of review, 3 items were moved to other item banks, and 6 new items were added. Finally, 2 additional items were added from the related Traumatic Brain Injury - Quality of Life study.

With the exception of the 5 items originally from Neuro-QOL which already underwent cognitive debriefing, this refined set of SCI-QOL Self-esteem items was then evaluated with individuals with SCI during structured cognitive debriefing interviews,^29^ in which respondents were asked to answer each item, then describe the process they used to come up with their answer and relate whether they perceived anything to be confusing, unclear, or derogatory, or whether they thought any items could be better phrased. One item was modified and no items were deleted based on cognitive interviewing. After this phase, the set of 30 items was reviewed for translatability (for method, please see Eremenco *et al*.^30^) and reading level (using the Lexile framework^31^). Slight modifications were made to 6 items after the translatability and cultural review. For example, the item ‘I was self-conscious about performing new tasks’ was changed to ‘Because of my injury, I worried about performing tasks in front of other people,’ since translation to the word ‘*self-conscious*’ would not be possible in this context. All items were written at the 5th grade reading level.

### Calibration study participants and data collection procedures

As a part of a large-scale multisite item calibration study (sites included the Kessler Foundation, University of Michigan, Rehabilitation Institute of Chicago, University of Washington, Craig Hospital and the James J. Peters/Bronx Veterans Administration hospital), we administered the 23 self-esteem items along with other item pools reflecting different HRQOL subdomains to a sample of people with SCI.

The calibration sample included 717 participants with SCI. Inclusion criteria were 18 years of age and older, ability to read and understand English, and had a medically documented traumatic SCI. The sample was stratified by level (paraplegia versus tetraplegia), completeness of injury (complete vs. incomplete), and time since injury (<1 year, 1–3 years, and >3 years) to ensure that the final sample was a heterogeneous sample of individuals with SCI. Each participant's diagnosis was confirmed by medical record review; neurologic level was documented by their most recent American Spinal Injury Association Impairment Scale (AIS) rating.^32^ All items were presented in a structured interview to participants in person or over the phone. The methodology for this study is presented in detail in Tulsky *et al*.^26^

### Data analyses

Analysis involved confirmation of construct unidimensionality, use of a graded-response IRT model to calibrate item parameters, and examination of differential item functioning (DIF). We used confirmatory factor analyses (CFA) to determine if our items conformed to a unidimensional model. Acceptable model fit indices were: CFI > 0.90, RMSEA < 0.08, good; CFI > 0.95, RMSEA < 0.06, excellent). Calibration was performed using iterative methods to reduce the item pool and obtain the best-fitting item parameters that would best allow estimation of a participant's standing on a trait of self-esteem. With each successive analytic iteration, we identified poorly fitting items by examining item fit to the graded response IRT model,^33^ DIF, local dependence between items (residual correlations >|0.15|), and significant loadings on the single factor (values >0.30). We then removed these items from the item pool and repeated the analytic steps. Once an acceptable solution was reached with CFA statistics that supported a unidimensional model, and all items showing misfit to the model or DIF were removed, the final IRT parameters were utilized to develop computer adaptive test (CAT) algorithms for the Self-esteem item bank. The CAT was programmed on the Assessment Center website (http://www.assessmentcenter.net) and can be administered directly from there. The item parameters were also used to select items for a static short form which can also be downloaded as a PDF from the Assessment Center website. Tulsky *et al.* within this special issue described the detailed methodology and data analysis plan.^26^

## Results

### Participant characteristics

Self-esteem items and other item pools were administered to a calibration sample of 717 individuals with SCI. Demographic and injury characteristics are summarized in Table 1. Please see the Tulsky *et al.* introductory article within this special issue for additional details on the calibration sample, including education, income and mechanism of injury.^25^

**Table 1:** Participant characteristics

Variable	Emotional domain sample, *N* = 717; Mean (SD), *N* (%)
Age (years)	43.0 (15.3)
Age at injury (years)	36.1 (16.8)
Sex	
Male	559 (78%)
Female	158 (22%)
Ethnicity	
Hispanic	82 (11%)
Non-Hispanic	631 (88%)
Unknown/Not reported	4 (1%)
Race	
Caucasian	505 (70%)
African-American	125 (17%)
Asian	8 (1%)
American Indian/Alaska Native or Native Hawaiian/Pacific Islander	7 (1%)
More than one race	9 (1%)
Other	50 (7%)
Unknown/Not reported	13 (2%)
Time since injury	7.1 (10.0)
<1 year post injury	196 (27%)
1–3 years post injury	186 (26%)
>3 years post injury	335 (47%)
Diagnosis	
Paraplegia complete	182 (25%)
Paraplegia incomplete	143 (20%)
Tetraplegia complete	157 (22%)
Tetraplegia incomplete	231 (33%)
Unknown	4 (1%)

### Preliminary analysis and item removal

Of the original 30 items that were tested, 7 were removed for the following reasons (reasons for removal were not mutually exclusive): local item dependence (5 items), low item-total correlations (5 items), and misfit (significant S-X^2^ test; 2 items). For the final 23 retained items, internal consistency was *α* = 0.950 and item/total correlations ranged from 0.50 to 0.81. All of the items but one had more than 25% of the sample selecting category of ‘5’ (Always, or Never for reversed item). Data from one case was deleted due to excessive missing data. No items had sparse data (i.e. <5 responses) in any category. Two items had a category inversion with the average raw score for persons selecting category ‘2’ (Often) were lower than the average for person selecting category ‘1’ (Always). No further items were removed.

### Dimensionality

Using CFA, a unidimensional model was observed (CFI = 0.946; RMSEA = 0.087). *R*^2^ values for 20 of the items were greater than 0.40 and 3 items were less than 0.40. In terms of local dependence, no item pairs were identified (i.e. residual correlations >|0.20|). Eigenvalue ratio (first to second) was 10.5.

### IRT parameter estimation and model fit

Slopes ranged from 1.28 to 3.74; thresholds ranged from −3.93 to 1.38 (see Table 2).

**Table 2:** Self-esteem items and item bank parameters

			Item response theory calibration statistics				
Item ID	Response set	Item stem	Slope	Threshold 1	Threshold 2	Threshold 3	Threshold 4
**SelfE_13**	**a**	**I felt bad about myself.**	**2.88369**	**−2.09421**	**−1.39942**	**−0.50925**	**0.18300**
SelfE_10	a	I've been unhappy with the person I've become since my injury.	2.36523	−2.16006	−1.50417	−0.71821	−0.04493
**AltStem_NQSTG07**	a	**Because of my injury, I felt embarrassed in social situations.**	**2.58943**	**−2.12140**	**−1.45995**	**−0.58047**	**0.04373**
**AltStem_NQSTG12**	a	**I am unhappy about how my injury affected my appearance.**	**2.20770**	**−1.46038**	**−0.87027**	**0.02849**	**0.62986**
SelfE_27	b	I had high self-esteem.	2.03773	−1.97060	−1.17425	−0.10977	0.85562
SelfE_9	a	Because of my injury, I worried about performing tasks in front of other people.	2.12675	−2.12899	−1.35373	−0.41048	0.20603
SelfE_33	b	I felt good about myself.	2.46332	−2.36479	−1.46769	−0.39789	0.62883
SelfE_15	a	I felt insecure.	2.63998	−2.33476	−1.51394	−0.52884	0.20976
**SelfE_25**	a	**I felt invisible to other people.**	**1.53538**	**−3.93586**	**−2.73207**	**−1.12792**	**−0.31595**
Self_23	a	I was unhappy with the way my clothes fit me.	1.28351	−2.42998	−1.57067	−0.38942	0.33344
SelfE_8	a	I felt it was difficult to achieve goals I set for myself.	1.87950	−2.13671	−1.27073	0.01359	0.72910
SelfE_32	b	I was comfortable with myself.	2.14478	−1.97928	−1.27433	−0.30659	0.65866
SelfE_17	b	I felt attractive.	1.56495	−1.74028	−0.73346	0.55838	1.38189
SelfE_18	a	I was ashamed of my injury.	2.48201	−2.24720	−1.77481	−1.04727	−0.55576
SelfE_26	a	I felt embarrassed about needing a bowel or bladder management program.	1.68809	−2.09160	−1.49499	−0.59452	−0.02095
SelfE_22	a	I was unhappy with changes in my posture.	1.38021	−2.34324	−1.22061	−0.24168	0.38506
**AltStem_NQSTG17**	a	**I felt embarrassed about my physical limitations.**	**2.65439**	**−2.09386**	**−1.46426**	**−0.50970**	**0.04897**
**SelfE_7**	a	**Because of my injury, I was unhappy with who I am.**	**3.33880**	**−2.03852**	**−1.30383**	**−0.54243**	**−0.05638**
**SelfE_12**	a	**I felt inferior to my friends or family.**	**2.64976**	**−2.32666**	**−1.70477**	**−0.84988**	**−0.32136**
AltStem_NQSTG20	a	I tended to blame myself for my problems.	1.45220	−2.24114	−1.47577	−0.47925	0.19288
**SelfE_24**	a	**I felt I was no longer a "whole person".**	**2.68332**	**−1.73861**	**−1.23531**	**−0.50265**	**−0.00837**
SelfE_14	a	I had poor self-esteem.	3.74315	−2.07225	−1.34651	−0.52923	0.06185
SelfE_20	a	I always compared myself to people who have not been injured.	1.78641	−2.10016	−1.40414	−0.44759	0.17593

NOTE: Context for all self-esteem items is ‘Lately’; Response set ‘a’ was: 5 = Never/4 = Rarely/3 = Sometimes/2 = Often/1 = Always; Response set ‘b’ is 1 = Never/2 = Rarely/3 = Sometimes/4 = Often/5 = Always.

**Items in bold** represent short form selections. Items and parameters copyright © 2015 David Tulsky and Kessler Foundation. All Rights Reserved. Scales should be accessed and used through the corresponding author or http://www.assessmentcenter.net. Do not modify items without permission from the copyright holder.

**Figure 1:**
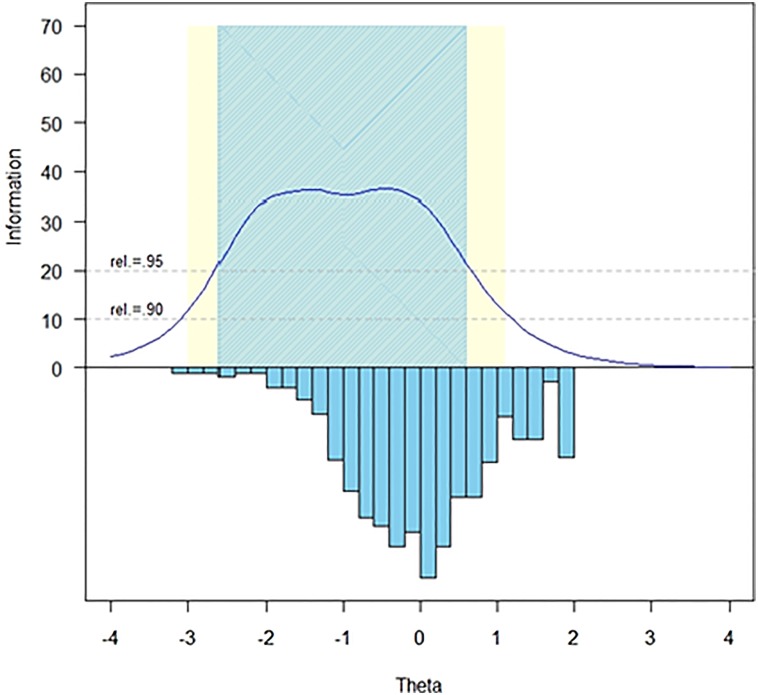
SCI-QOL Self-esteem Item Bank Information and Precision

The measurement precision in the theta range between −2.7 and 0.7 is roughly equivalent to a classical reliability of 0.95 or better (Fig. 1).

**Figure 2:**
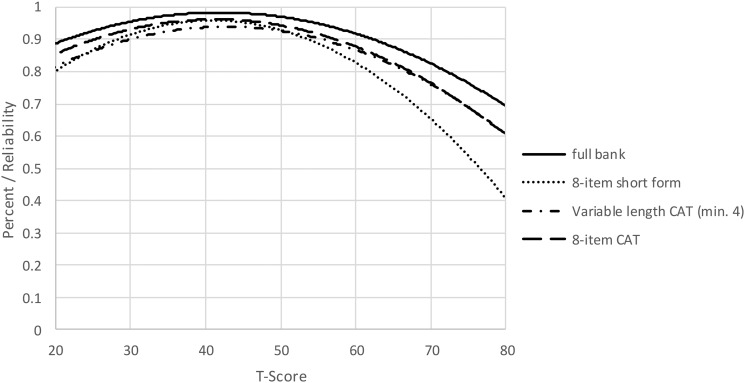
Measurement Reliability by T-Score and Assessment Method. Note: CAT, Computer Adaptive Testing, which was simulated from calibration data using Firestar^36^

The S-X^2^ model fit statistics were examined using the IRTFIT macro program. All items had adequate or better model fit statistics (P < 0.05), with marginal reliability equal to 0.946 and no item pairs were flagged (|*r*|> = 0.4) for local dependence (Fig. 2).

### Differential item functioning

DIF was examined using *lordif*^34^ for six categories: age (≤49 vs. ≥50), sex (male *n* = 559 vs. female *n* = 158), education (some college and lower *n* = 523 vs. college degree and above *n* = 194), diagnosis (tetraplegia *n* = 388 vs. paraplegia *n* = 325), injury severity (incomplete *n* = 374 vs. complete *n* = 339), and time post injury (<1 year *n* = 196 vs. >1 year *n* = 521). Items were flagged for possible DIF when the probability associated with the *χ*^2^ test was <0.01 and the effect size measures (McFadden's pseudo *R*^2^) >0.02, which is a small but non-negligible effect. Overall, 11 items were flagged for DIF in at least one category based on the *χ*^2^ test; however, when the effect size measures were examined, the DIF was negligible and all 23 items were retained in the final, calibrated item bank.

### Short form selection and mode of administration

Once the SCI-QOL Self-esteem item bank was finalized, all items and parameters were programmed into the Assessment Center^SM^ ^35^ platform and the bank is now freely available as a CAT. Since the purpose of calibrating items using IRT is that only a subset of items needs to be administered from a given bank in order to estimate an individual's score, there is flexibility as to how the items are selected and administered. On the Assessment Center platform, the CAT administration parameters can be modified to reduce standard error variance (e.g. maximize reliability), or to reduce test burden. There is also a predetermined static short form that can be downloaded. Finally, the individual items are present and could be selected if the end user wanted to administer a specific item. These administration options are reviewed below.

The SCI-QOL utilizes the same default CAT discontinue criteria as PROMIS; namely, the CAT minimum number of items to administer is four and the maximum is 12 with a maximum standard error of 0.3. In other words, in the default settings, the CAT will always administer at least 4 items, then will discontinue when the standard error of the individual's score estimate drops below 0.3 or a maximum of 12 items is reached (and the standard error variance criterion cannot be met).

Alternatively, the user could change the ‘discontinue criteria’ of the CAT so that it will administer additional items and obtain a more precise assessment of functioning. For instance, if the user selected an option that the CAT administers a minimum of 8 items before discontinuing, a lengthier test would be administered, but a more reliable score will be obtained. In some cases, greater precision over test burden is desirable based on factors such as resource allocation where specificity is critical.

However, in some cases it is neither possible (e.g. internet unavailable) or practical (e.g. laptop/tablet computer equipment beyond budget of project) to administer items via CAT. To address this need, the self-esteem and other SCI-QOL item banks are also available as short forms. The project investigators utilized psychometric and clinical input to develop a fixed, 8-item short form version of the self-esteem item bank. The goal of the short form selection process was to include the most informative items across a wide range of ‘difficulty’, or amount of the underlying trait. Since all items are calibrated on the same metric, scores on the short form are directly comparable to those on the CAT or full item bank. The correlation of the short form and various CATs with the full bank are given in Table 3. Short forms may be administered directly within Assessment Center, or may be downloaded for administration by paper and pencil or an alternate data capture platform or system. Individual investigators or clinicians could also develop additional, custom short forms, which could then be scored on the same IRT-based metric with the help of a psychometrician.

**Table 3:** Accuracy of variable- and fixed-length CAT and 8-item short form: correlations with full-bank score

Mode	*N*	# Items admin						Corr. w/Full-bank score
		Mean	SD	Min	Max	%Min	%Max	
Variable-length CAT (min 4)	716	6.78	3.17	4	12	40.4	20.7	0.974
Variable-length CAT (min 8)	716	8.98	1.64	8	12	71.0	20.7	0.983
8-Item fixed-length CAT	716	8	0	8	8	n/a	n/a	0.975
8-Item short form	716	8	0	8	8	n/a	n/a	0.953

To determine the degree of measurement precision and error for these assessments, we compared the reliability of the full bank, 8-item short form, and variable-length CAT with the default minimum of 4 items. Table 4 presents the mean, standard deviation, range, and standard error ranges for the various administration modes. Additionally, reliability curves for the full bank, short form, variable length CAT (minimum of 4 items) and fixed-length CAT (8 items) may be found in Fig. 2.

**Table 4:** Breadth of coverage for variable length CAT, fixed length CAT, 8-item short form, and full item bank

Mode	*N*	T score				Standard error	
		Mean ± SD	Range	% Ceiling	% Floor	Mean ± SD	Range
Variable-length CAT (min 4)	716	50.45 ± 9.57	18.86–70.07	5.03%	0.14%	0.307 ± 0.067	0.248–0.529
Variable-length CAT (min 8)	716	50.45 ± 9.58	18.86–70.07	5.03%	0.14%	0.275 ± 0.083	0.190–0.529
8-Item fixed-length CAT	716	50.48 ± 9.58	20.16–68.90	6.42%	0.56%	0.286 ± 0.096	0.190–0.543
8-Item short form	716	50.25 ± 9.05	21.30–63.90	14.94%	0.28%	0.327 ± 0.116	0.21–0.57
Full bank	716	50.36 ± 9.65	18.50–70.80	4.33%	0.14%	0.216 ± 0.089	0.140–0.510

When we instead compared the reliability of a CAT that was either fixed to 8 items, or a variable-length CAT with a minimum of 8 items, CAT values for both reliability (Fig. 2, reliability curves figure) and precision (Table 4, Breadth of coverage table) demonstrated improvement over the short form values.

### Scoring

SCI-QOL Self-esteem scores are standardized on a T-metric, with a mean of 50 and a standard deviation of 10; this is based on the SCI-QOL calibration data; that is, a mean of 50 reflects the mean of an SCI population rather than the general population. All CAT administrations of the SCI-QOL Self-esteem item bank is automatically scored by Assessment Center. When administering the short form, whether via Assessment Center, paper and pencil, or another data capture platform, an individual must complete all 8 component items in order to receive a score. The raw score for the short form is computed by simply summing the response scores for the individual component items. The T-score and associated standard error for each raw score value is given in Table 5.

**Table 5:** T-score lookup table for SCI-QOL Self-esteem SF8a

Raw score	Scaled score	Standard error
8	19.6	4.5
9	21.9	4.0
10	24.0	3.6
11	26.1	3.2
12	27.7	3.0
13	29.1	2.8
14	30.4	2.7
15	31.6	2.7
16	32.7	2.6
17	33.8	2.6
18	34.8	2.6
19	35.8	2.6
20	36.8	2.6
21	37.7	2.6
22	38.7	2.6
23	39.6	2.6
24	40.6	2.6
25	41.5	2.6
26	42.4	2.6
27	43.3	2.6
28	44.3	2.6
29	45.2	2.6
30	46.2	2.6
31	47.1	2.6
32	48.1	2.6
33	49.2	2.7
34	50.3	2.8
35	51.6	2.9
36	53.0	3.2
37	54.6	3.4
38	56.7	3.9
39	58.7	4.1
40	63.9	5.7

### Reliability

As a part of the reliability study described in the Tulsky *et al*.^26^ methods paper in this issue, we compared Self-esteem scores at Baseline with those from the 1–2 week retest assessment. In a sample of 245 individuals with SCI, Pearson's *r* = 0.84 and ICC (2,1) = 0.84 (95% CI = 0.80 to 0.88).

## Discussion

The SCI-QOL Self-esteem item bank is unique among cutting-edge measurement systems such as Neuro-QOL or the Patient Reported Outcomes Measurement Information System (PROMIS) for two primary reasons. First, the construct of self-esteem is not included in PROMIS, Neuro-QOL, or any other IRT-based assessments of health outcomes. Second, and perhaps most importantly, the SCI-QOL Self-esteem item bank was developed from the ground up with input from individuals with SCI, to the extent that many items were based directly off of verbatim interview or focus group quotes. The groundedness of the included items was continually assessed throughout measurement development, through the use of cognitive debriefing interviews and large scale calibration testing, both with individuals with SCI and with clinicians who specialize in SCI. As a result, this new item bank captures self-esteem as it specifically relates to SCI.

Rosenberg *et al*. differentiate global self-esteem from specific self-esteem, with the latter a better predictor of behavior and the former of psychological well-being.^36^ The final SCI-QOL Self-esteem item bank represents global self-esteem by tapping into the psychological and, more specifically, affective, dimensions of self-esteem. Items, primarily drawn from verbatim comments during focus groups, are characterized by words such as ‘unhappy’, ‘ashamed’, or ‘embarrassed’. Future work should include differentiating the item bank from psychological measures of depression or anxiety, for example, and validation as a measure of global self-esteem. In the long-term, the SCI-QOL Self-esteem item bank can promote the development of conceptual models of self-esteem in SCI.

The use of IRT to calibrate the SCI-QOL Self-esteem items has yielded several administration options, including short forms and CAT. If a user's goal is to optimize reliability, especially at the ceiling and floor of the distribution, we would recommend administering the Self-esteem item bank as a CAT with a minimum of 8 items. It is worth noting also that the CAT with the default parameters (minimum 4 items) performs nearly as well as the 8-item SF while reducing respondent burden. In a situation where brevity of assessment is preferred over a modest increase in reliability, it would make the most sense to administer a CAT with the default parameters. In cases where it may not be feasible or practical to administer items via CAT/Assessment Center, or if having participants answer the same subset of 8 items is necessary to answer a given research question, we would recommend short form administration. An additional administration option is to administer both the CAT and any short form items not included in the CAT by using the ‘no duplicates’ option in Assessment Center. In this way, the user could optimize reliability and have the option of directly comparing individuals' responses on specific items to each other or to themselves over time. Future directions include evaluation of self-esteem as a moderator of a variety of outcomes following SCI, most notably emotional outcomes such as depression and anxiety.

The flexibility of methods to administer the SCI-QOL Self-esteem item bank also provides scientists and clinicians with an efficient and accessible way to integrate the measurement of self-esteem that is specifically relevant to SCI into research and, ultimately, clinical practice. Previous literature on self-esteem and SCI points to the important relationship of this concept to psychological well-being. Toward this end, research is needed to examine the validity of this new measure in the context of SCI. For example, studies are needed to determine how self-esteem changes over time and its role in adaptation and adjustment to injury. It will also be important to determine whether self-esteem predicts other important outcomes such as quality of life, functional impairment, and self-care and the degree to which self-esteem is amenable to change.

## Conclusion

The final SCI-QOL Self-esteem item bank contains 23 IRT-calibrated items. Due to the flexibility of IRT-based measures, the use of CATs is also possible with this item bank, which enables researchers and clinicians to administer only the most precise and informative items based on an individual's responses. This has implications for the use of such innovative applications in symptom monitoring and self-management in post-acute care settings. To the best of our knowledge, this is the first time that a patient-centered, modern measurement theory derived approach has been used to develop and test a self-esteem, self-reported measurement tool specifically designed for individuals with SCI. Our formative development work using focus groups and interviews strengthened our understanding of self-esteem in the context of SCI and its utility and importance with this population. This, coupled with the paucity of such a measurement tool in the extant rehabilitation medicine literature, makes this effort an important first step towards a greater understanding of the role of self-esteem and related factors in the short and long term adaptation to SCI and its subsequent psychosocial sequelae.

## Disclaimer statements

**Contributors** All authors have contributed significantly to the design, analysis and writing of this manuscript.

**Funding** This study was supported by grant #5R01HD054659 from the National Institutes of Health – Eunice Kennedy Shriver National Institute of Child Health and Human Development/National Center on Medical Rehabilitation Research and the National Institute on Neurological Disorders and Stroke.

**Conflicts of interest** The contents represent original work and have not been published elsewhere. No commercial party having a direct financial interest in the results of the research supporting this article has or will confer a benefit upon the authors or upon any organization with which the authors are associated. All SCI-QOL items and parameters are © 2015 David Tulsky and Kessler Foundation. All rights reserved. All SCI-QOL items originally from Neuro-QOL are © 2008–2013 David Cella on behalf of the National Institute for Neurological Disorders and Stroke (NINDS). All items are freely available to the public via the Assessment Center platform (http://www.assessmentcenter.net). There are currently no plans for Dr. Tulsky, Kessler Foundation, or the NINDS to profit from the use of the copyrighted material.

**Ethics approval** The Institutional Review Board at each site reviewed and approved this project.
